# Death of Leukemia Cells and Platelets Induced by 3,3′-Dihydroxy-4,5-Dimethoxybibenzyl Is Mediated by p38 Mitogen-Activated Protein Kinase Pathway

**DOI:** 10.3390/molecules30142965

**Published:** 2025-07-15

**Authors:** Natalia Rukoyatkina, Tatyana Sokolova, Nikita Pronin, Andrei Whaley, Anastasiia O. Whaley, Stepan Gambaryan

**Affiliations:** 1 Sechenov Institute of Evolutionary Physiology and Biochemistry of the Russian Academy of Sciences, Saint Petersburg 194223, Russia; natalia.rukoyatkina@gmail.com (N.R.); sokolt1956@gmail.com (T.S.); nikitapronin.platelets@gmail.com (N.P.); 2 Department of Pharmacognosy, Saint Petersburg State Chemical and Pharmaceutical University, Saint Petersburg 197022, Russia; 9968639@gmail.com (A.W.); anastasiya.ponkratova@yandex.ru (A.O.W.)

**Keywords:** bibenzyls, platelets, MOLT-3 cells, p38 mitogen-activated protein kinase (p38 MAPK), cyclic adenosine monophosphate (cAMP), cyclic guanosine monophosphate (cGMP), protein kinase A (PKA), protein kinase G (PKG)

## Abstract

Bibenzyls are now recognized as compounds for use in cancer therapy, and many molecules from the bibenzyl group have shown promising anticancer activity; therefore, the characterization of new bibenzyls with strong biological activity is important for developing new anticancer drugs. In this study, we compared the effects of three bibenzyls (3,3′-dihydroxy-4,5-dimethoxybibenzyl, 3,5-dihydroxy-4-methoxybibenzyl and 3,5,3′-trihydroxy-4-methoxybibenzyl) isolated from *Empetrum nigrum* and erianin on platelets and the MOLT-3 T-lymphoblast cell line. Among the studied bibenzyls, 3,3′-dihydroxy-4,5-dimethoxybibenzyl significantly reduced the viability of MOLT-3 cells and platelets and induced strong phosphatidylserine (PS) surface exposure. We showed that 3,3′-dihydroxy-4,5-dimethoxybibenzyl induced the death of MOLT-3 cells and platelets, which was not mediated by apoptosis, pyroptosis, necroptosis, autophagy, or calpain-dependent pathways, and that the p38 MAP kinase pathways are at least partly involved in the activity of 3,3′-dihydroxy-4,5-dimethoxybibenzyl. In conclusion, our data show that 3,3′-dihydroxy-4,5-dimethoxybibenzyl could be a promising candidate for future analysis as an anticancer drug.

## 1. Introduction

Natural products demonstrate high potential against various diseases such as cancer, anxiety, inflammation, viral infections, thrombosis, and others [1,2,3,4]. They can also be applied in combination with therapeutic drugs for the treatment of different diseases [5]. More than 60% of currently used drugs are obtained from natural sources, and many of them, such as paclitaxel, colchicine, bergenin, curcumin, coumarins, and combretastatin, are used as anticancer drugs [6]. Among many other natural products, bibenzyls with a very simple skeleton are now recognized as molecules for use in cancer therapy, and many compounds from the bibenzyl group have shown promising anticancer activity in different cancer cell lines. For example, among 89 small molecule bibenzyls isolated from the *Dendrobium* genus, 23 possess anticancer activity [7]. The bibenzyl erianin showed the highest potential for clinical use. It inhibits different cancer cells at nanomolar concentrations [8,9]. However, in oral squamous cell carcinoma cells, erianin induced apoptosis at a micromolar level [10].

Cancer patients have an increased risk of both thrombosis and thrombocytopenia [11]. The development of thrombosis is closely related to the poor prognosis of cancer patients [12]. Thrombocytopenia is a frequent adverse effect of chemotherapy and, in addition to increasing the bleeding risk, it limits the dose and frequency of cancer therapy. Platelets are responsible for both thrombotic complications and thrombocytopenia. Several widely used anticancer drugs, like navitoclax, oxaliplatin, and some others, act through the activation of apoptosis in cancer cells, and thrombocytopenia, as a result of platelet apoptosis induced by these drugs, is a common consequence for the patients treated with these anticancer agents [13,14,15]. Bibenzyls are now recognized as promising chemicals with high potential to develop new anticancer drugs; therefore, it is very important to investigate the effects of bibenzyls on different cancer cell lines. Since many anticancer drugs can have strong effects on platelet function and viability, it is also important to simultaneously analyze platelets under similar experimental conditions. In the literature, we found only very limited information concerning the effects of bibenzyls on platelets [7,16,17,18]. The inhibition of platelet aggregation by *Dendrobium* secondary metabolites—trigonopol A [18], moscatilin [16,17] and gigantol [17]—has been described.

**Figure 1:**
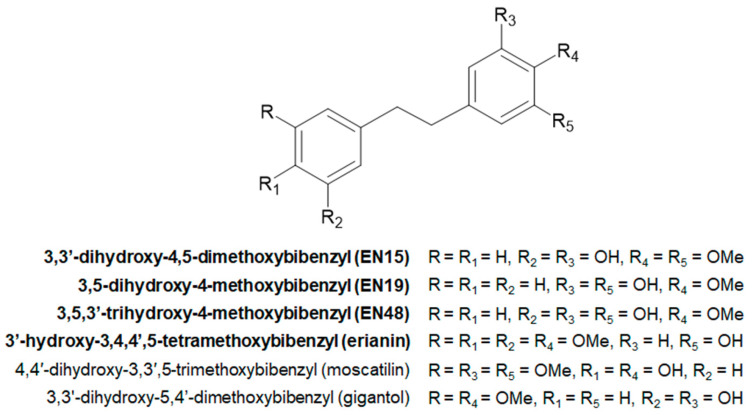
The bibenzyl scaffold and structures of six derivatives. The compounds investigated are marked in **bold**.

Since plants from the genus *Dendrobium* are epiphytic and lithophytic orchids that are distributed predominantly in southeast Asia [19], we searched for another botanical source of bibenzyls. The evergreen shrub *Empetrum nigrum* from the *Ericaceae* family was found to be the best research candidate due to its circumboreal distribution, rich diversity of bibenzyl secondary metabolites and having a variety of ethnopharmacological/pharmacological data associated with it [20]. As a result of previous research, we isolated three bibenzyls: 3,3′-dihydroxy-4,5-dimethoxybibenzyl (EN15), 3,5-dihydroxy-4-methoxybibenzyl (EN19), and 3,5,3′-trihydroxy-4-methoxybibenzyl (EN48) from the plant *Empetrum nigrum*, which differ from each other by the position and number of methoxy groups in the molecule [21]. One of these compounds, 3,3′-dihydroxy-4,5-dimethoxybibenzyl, significantly reduced platelet viability [22], and the other two inhibited thromboxane synthase activity without inhibition of thrombin-induced integrin αIIbβ3 activation [23]. The bibenzyl scaffold, the structures of isolated compounds and other bibenzyls possessing anticancer activity (including erianin) are presented in Figure 1.

In the presented study, we compared the effects of three previously characterized bibenzyls and erianin on platelets and the MOLT-3 T-lymphoblast cell line and showed that 3,3′-dihydroxy-4,5-dimethoxybibenzyl (EN15) had the strongest effect on MOLT-3 cells and platelets. Additionally, we demonstrated that this bibenzyl acts through the p38 mitogen-activated protein kinase pathway.

## 2. Results

### 2.1. Effect of Bibenzyls on Phosphatidylserine Surface Exposure and Viability of MOLT-3 Cells and Platelets

**Figure 2:**
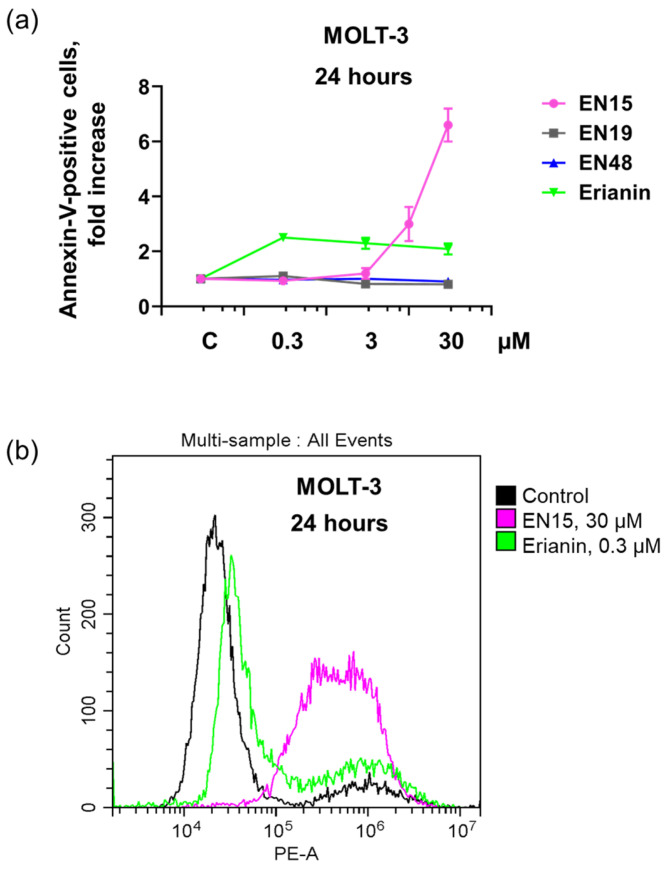
Effect of EN15 on PS exposure in MOLT-3 cells is concentration-dependent, in contrast to other bibenzyls. (**a**) MOLT-3 cells were incubated with the indicated concentrations of EN15 and EN19/EN48/erianin, for 24 h at 37 °C. Then PS surface expression was analyzed by flow cytometry of annexin-V-PE binding. Data are presented as means ± SD, non-parametric Mann–Whitney test, *n* = 6. (**b**) The representative histogram (from six independent experiments) demonstrates the change in annexin-V binding of cells incubated with erianin (0.3 µM, 24 h) or EN15 (30 µM, 24 h). C—control treated with corresponding concentration of DMSO.

Strong anticancer activity of several bibenzyls, including moscatilin [24], gigantol [25], and erianin [8,10,26] on different cancer cell lines is described. We compared the effect of four structurally related bibenzyl compounds (EN15, EN19, EN48, and erianin) on the T-lymphoblast cell line (MOLT-3). This cell line was chosen as an example of cancer cells on which the effects of bibenzyls were not studied. Over 24 h, EN19 and EN48 at certain concentrations (0.3–30 µM) did not significantly change PS surface exposure (annexin-V binding), whereas erianin (0.3 µM) increased annexin-V binding by 2.5 ± 0.34 times without stronger effects at higher concentrations. In contrast, EN15 dose dependently increased annexin-V binding, which at a concentration of 30 µM reached an increase of 6.2 ± 0.61-fold compared to control (Figure 2a,b).

**Figure 3:**
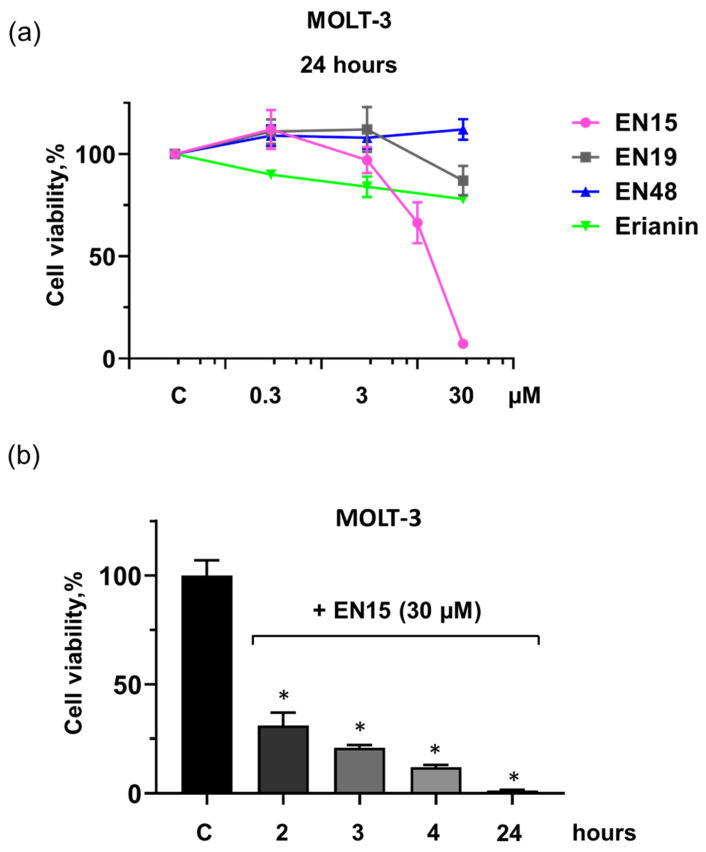
EN15 strongly inhibited the viability of MOLT-3 cells. (**a**) MOLT-3 cells were incubated with the indicated concentrations of EN15/EN19/EN48/erianin, for 24 h at 37 °C. MTT was added 2 h before the end of incubation. (**b**) MOLT-3 cells were incubated with EN15 (30 µM) for 2, 3, 4 or 24 h at 37 °C and MTT tests were used as in (**a**). Data are presented as means ± SD, non-parametric Mann–Whitney test, *n* = 6. *—*p* < 0.05 compared to control (C) taken as 100%. Control was treated with corresponding concentration of DMSO.

Similarly, EN15 decreased cell viability (MTT test) in a concentration-dependent manner to 7 ± 1.5%, whereas EN19 decreased viability only to 87 ± 7%, erianin (78 ± 2.4%) and EN48 did not change cell viability (Figure 3a). The decrease in cell viability induced by EN15 (30 µM) is time-dependent: after 2 h, it reached 31 ± 6%, after 3 h, 21 ± 1%, after 4 h, 12 ± 1%, and after 24 h, 1.3 ± 0.32%, compared to control (Figure 3b).

**Figure 4:**
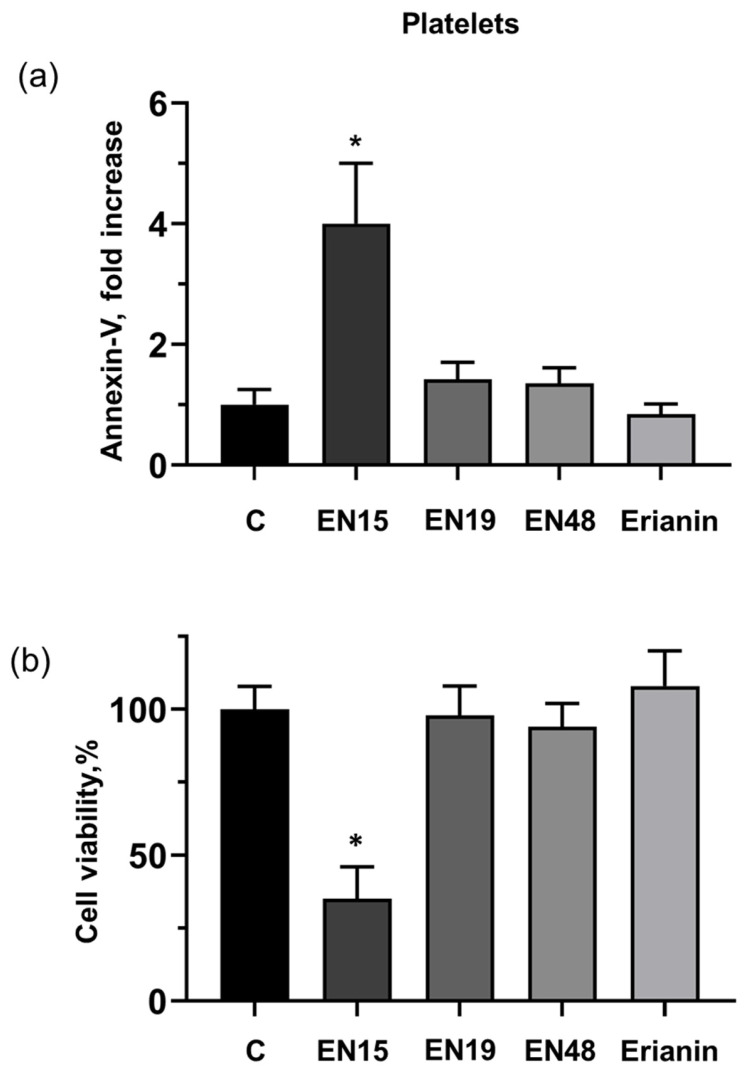
Only EN15 significantly induces PS exposure and decreases platelet viability. (**a**) Platelets were labelled with annexin-V-PE, then incubated with EN15, EN19, EN48 (60 µM) or erianin (30 µM) for 2 h at 37 °C. Then PS surface expression was analyzed by flow cytometry of annexin-V-PE binding. (**b**) Platelets were incubated with EN15 EN19, EN48 (60 µM) or erianin (30 µM) for 2 h at 37 °C. Platelet viability was analyzed by flow cytometry Calcein-AM staining. Data are presented as means ± SD, non-parametric Mann–Whitney test, *n* = 6. *—*p* < 0.05 compared to control (C) taken as 1 in A and 100% in B. Control was treated with corresponding concentration of DMSO.

Next, we evaluated the effects of these bibenzyls on platelet annexin-V binding and viability. Among the studied compounds, only EN15 (60 µM) strongly (3.9 ± 0.8 times) increased platelet PS surface exposure over two hours of incubation; EN19, EN48 and erianin did not induce significant annexin-V binding (Figure 4a). Accordingly, only EN15 significantly (35 ± 11%) reduced platelet viability measured by the Calcein test (Figure 4b).

**Figure 5:**
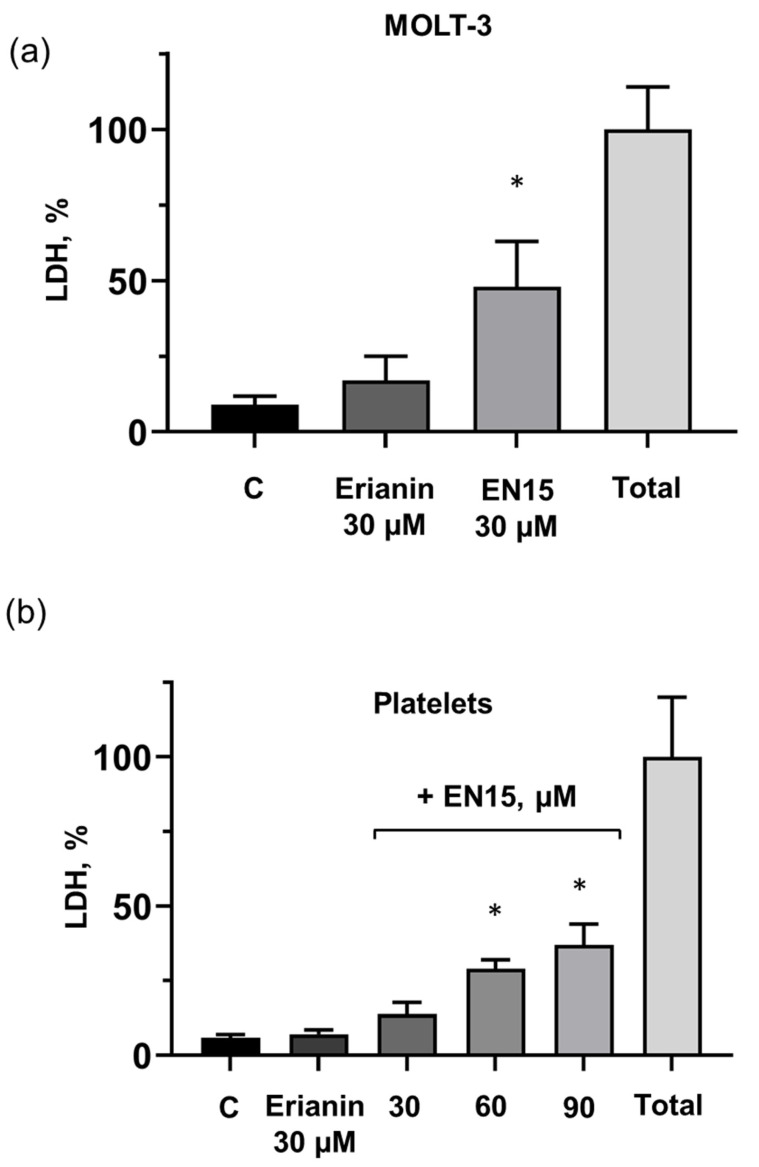
EN15 induces LDH release both in MOLT-3 cells (**a**) and platelets (**b**). LDH release was measured spectrophotometrically using the LDH-Vital kit at 340 nm by analyzing NADH reduction during pyruvate-lactate transformation. MOLT-3 cells were incubated with erianin (30 µM, 2 h), or EN15 (30 µM, 2 h). Platelets were incubated with erianin (30 µM), or EN15 with indicated concentrations for 2 h and LDH release was measured spectrophotometrically and calculated as a percentage of the total LDH concentration after lysis of cells by Triton-X 100 taken as 100%. Data are presented as means ± SD, non-parametric Mann–Whitney test, *n* = 6. *—*p* < 0.05 compared to control (C) sample treated with corresponding concentration of DMSO.

For evaluation of MOLT-3 cells and platelet viability, we used two different methods (MTT test for MOLT-3 cells and Calcein test for platelets). To prove that both these methods are appropriate for cells and platelets, we additionally used the LDH (lactate dehydrogenase) assay for both cell types as an indicator of cell viability. The measurements were carried out 2 h after drug application. Erianin only slightly (16 ± 8% compared to control 9 ± 2.8%) induced LDH release in MOLT-3 cells, whereas LDH release induced by EN15 achieved 48.6 ± 9.8% compared to the control (Figure 5a). Erianin had no effect on LDH release from platelets, and EN15 increased LDH release concentration dependently (Figure 5b). However, 30 µM of EN15 in 2 h induced 3.5 times more LDH release from MOLT-3 cells than from platelets: by 48 ± 10% and 13.8 ± 4%, respectively. Thus, our data showed that from the investigated bibenzyls, EN15 strongly affected MOLT-3 cells and platelet viability with a more pronounced effect on MOLT-3 cells.

### 2.2. EN15-Induced Death of MOLT-3 Cells and Platelets Is Not Mediated by Apoptosis, Pyroptosis, Necroptosis, or Autophagy Pathways

**Figure 6:**
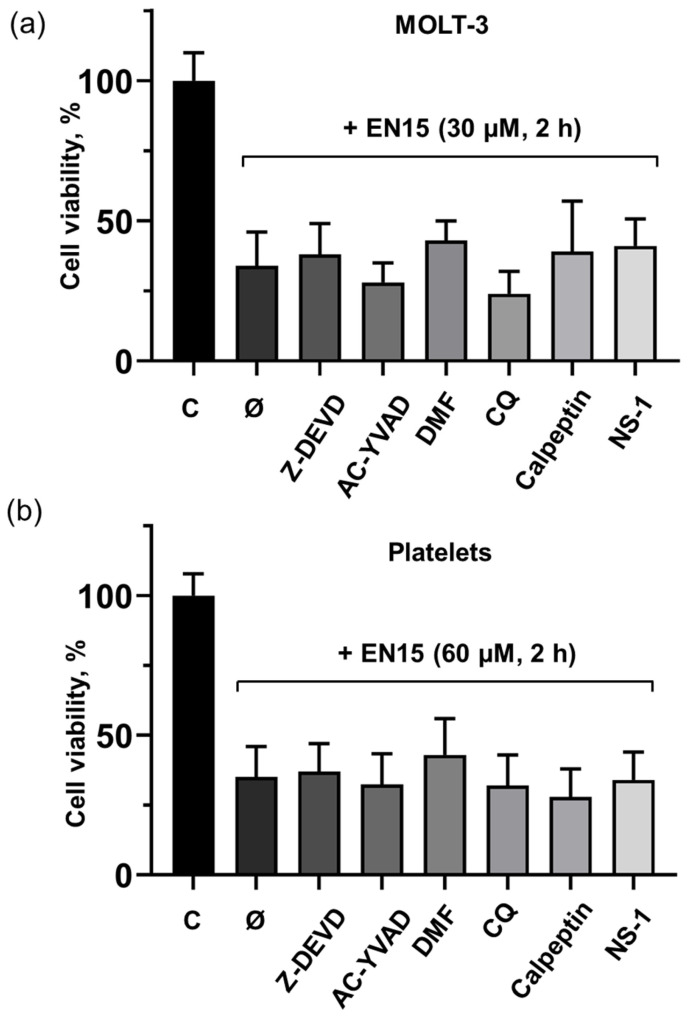
EN15 does not induce apoptosis, pyroptosis, necroptosis, autophagy, or calpain-dependent cell death in either MOLT-3 cells (**a**) or platelets (**b**). MOLT-3 cells and platelets were preincubated for 15 min at 37 °C with inhibitor of apoptosis Z-DEVD (50 µM), inhibitors of pyroptosis AC-YVAD (1 µM) and dimethylfumarate (DMF, 100 µM), inhibitor of autophagy chloroquine (CQ, 10 µM), calpeptin for calpain-dependent pathways (10 µM) or necrostatin-1 (NS-1, 10 µM) for necroptosis. (**a**) MOLT-3 cells were further incubated with EN15 (30 µM) and analyzed by MTT test as described in the methods. (**b**) Platelets were incubated with EN15 (60 µM) for 2 h at 37 °C and cell viability was analyzed by Calcein-AM staining. Data are presented as means ± SD, non-parametric Mann–Whitney test, *n* = 6. C—control treated with corresponding concentration of DMSO. Ø—EN15 sample without any cell death inhibitors.

Cell death may be mediated by different molecular mechanisms. To analyze which mechanisms mediate the death of these cells, we used specific inhibitors of pathways known to induce cell death. These experiments were carried out with the strongest inducer of cell death (EN15). MOLT-3 cells and platelets were preincubated with inhibitors of apoptosis (caspase 3 inhibitor, Z-DEVD); AC-YVAD, caspase 1 inhibitor and dimethylfumarate (both inhibit pyroptosis); necrostatin-1 for necroptosis; for autophagy (chloroquine); calpeptin for calpain-dependent pathways. None of the used inhibitors prevented the decrease in viability induced by EN15 in MOLT-3 cells (Figure 6a) and platelets (Figure 6b).

### 2.3. EN15 Induces Cells Death by Activation of p38 MAP Kinase Pathway

**Figure 7:**
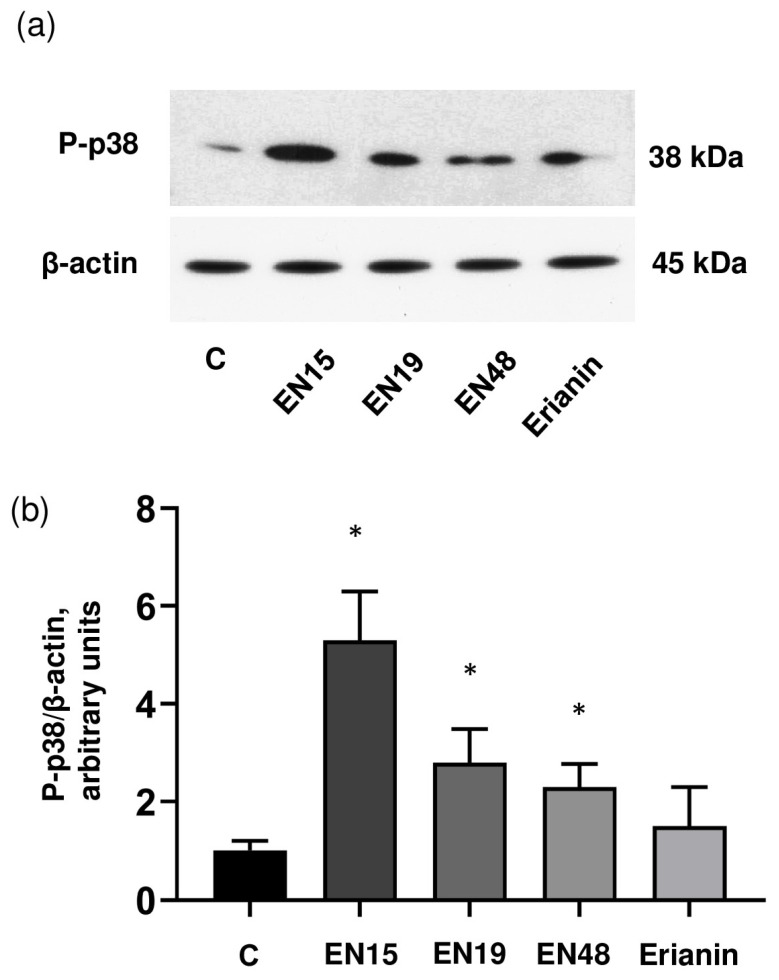
EN15 strongly induces p-38 MAP kinase phosphorylation. (**a**) Washed platelets were incubated with EN15/EN19/EN48 (60 µM) or erianin (30 µM) for 2 h at 37 °C, then processed for Western blot analysis. Actin served as a loading control. (**b**) Blots were scanned and analyzed by the NIH Image J software v1.54g. The intensity of the P-p38 signal was normalized to the actin signal. This ratio is relatively expressed to the ratio for the control, which was designated as 1. Data are presented as means ± SD of three separate experiments from three different donors. One-way ANOVA, Levene’s test *p* > 0.05 followed by Tukey’s HSD test. *—*p* < 0.05 compared to control (C) treated with corresponding concentration of DMSO. A representative blot from three separate experiments is shown. Full blots are presented in the Supplementary Materials (Supplementary Figure S2).

**Figure 8:**
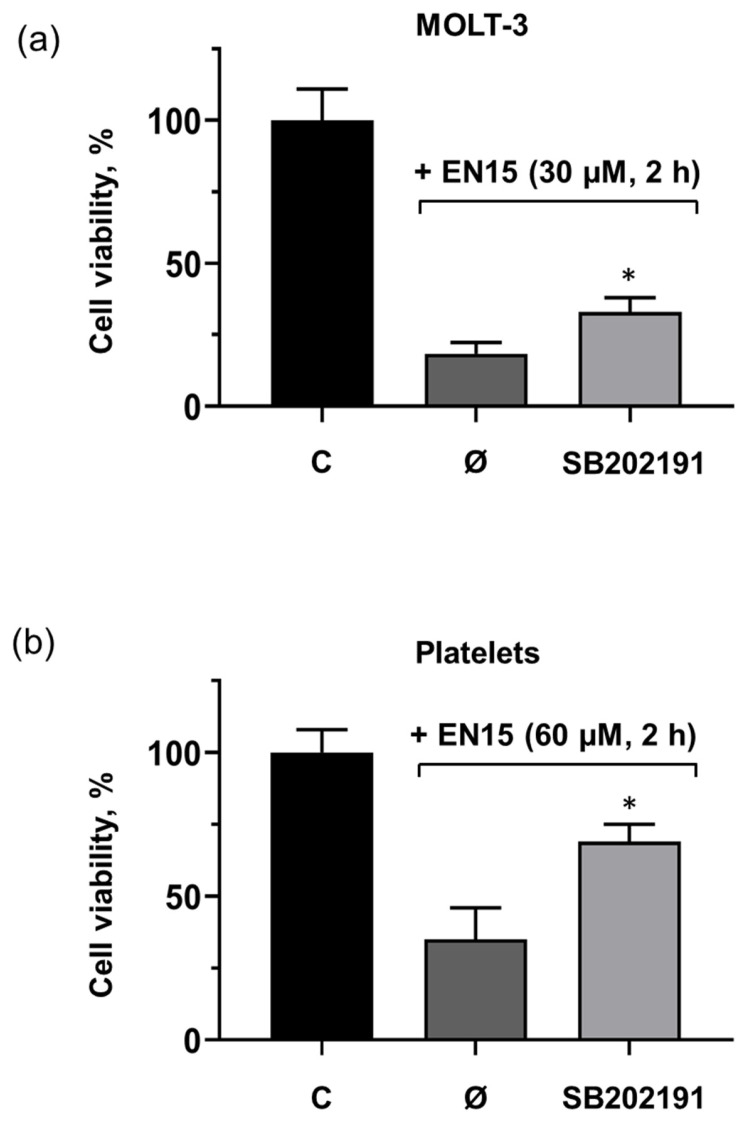
Inhibition of p38 MAP kinase significantly prevented EN15-reduced viability of MOLT-3 cells (**a**) and platelets (**b**). Cells were preincubated with SB202191 (20 µM, 15 min), further incubated with EN15 (30 µM, 2 h), and cell viability was analyzed by MTT test (MOLT-3 cells) or Calcein-AM staining (platelets) as described in the methods. Data are presented as means ± SD, non-parametric Mann–Whitney test, *n* = 6. *—*p* < 0.05 compared to EN15 sample without SB202191 (Ø). C—control treated with corresponding concentration of DMSO.

Signal transduction mediated by p38 MAP kinase plays a significant role in many pathways regulating cell survival, proliferation, differentiation, senescence, mitochondrial function, etc., [27,28]. In platelets, in addition to its role in different platelet activation pathways, p38 MAP kinase is involved in the regulation of ABT-737-induced platelet apoptosis [29]. Therefore, we tested whether EN15 activated (induced phosphorylation) the p38 MAP kinase pathways in platelets and whether an inhibitor of this kinase could prevent cell transformation. EN15 significantly (5.6 ± 0.7) increased p38 MAP kinase phosphorylation, whereas EN19 and EN48 only slightly induced phosphorylation (2.6 ± 0.3 and 2.4 ± 0.4-fold compared to control), and erianin did not significantly increase p38 MAP kinase phosphorylation (Figure 7a,b). The inhibitor of p38 MAP kinase (SB202191) significantly reduced EN15-induced MOLT-3 cell death (Figure 8a) and platelet (Figure 8b) death. Presented data indicate that EN15 altered MOLT-3 cells and platelet viability, at least partly, through activation of p38 MAP kinase pathways.

### 2.4. EN15 Inhibits Thrombin-Induced Platelet Activation

**Figure 9:**
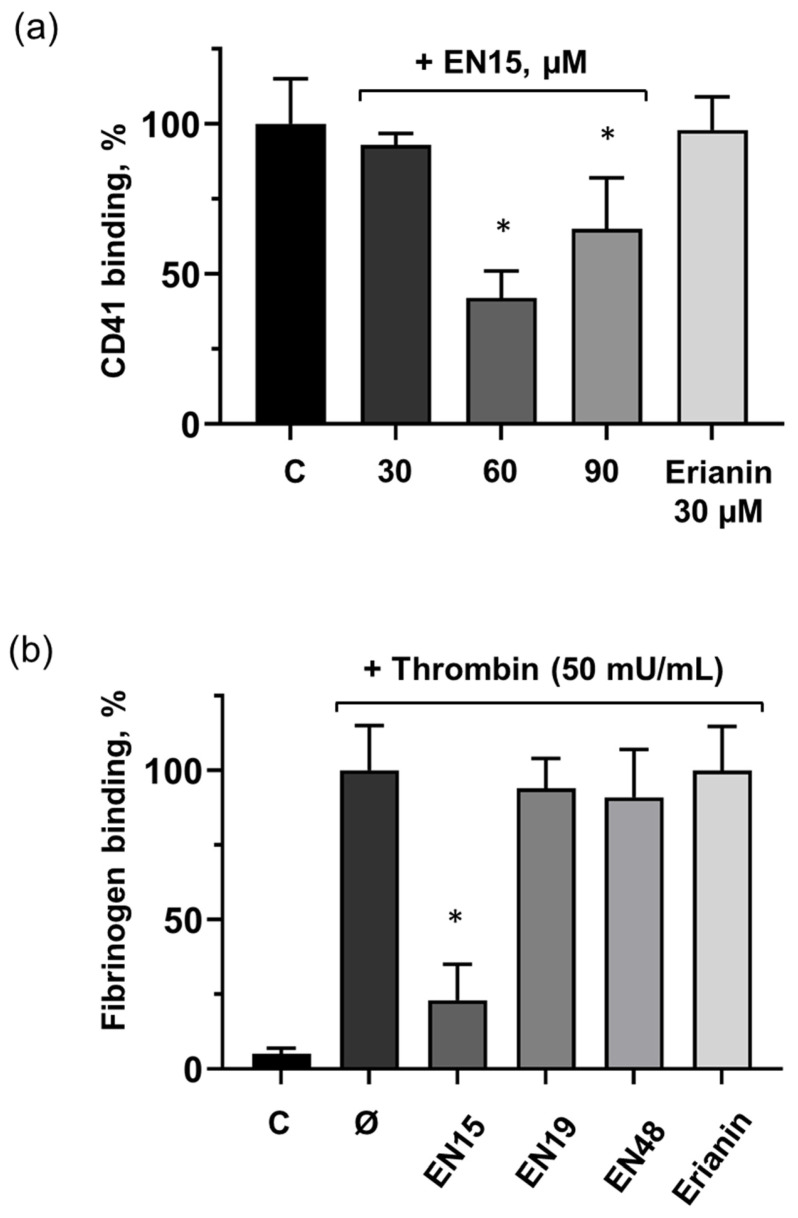
EN15 reduced αIIbβ3 integrin surface expression in platelets, which correlates with inhibition of thrombin-induced platelet activation. (**a**) Washed human platelets were incubated with EN15 (30, 60, 90 µM; 2 h, 37 °C) or erianin (30 µM; 2 h, 37 °C) and stained with CD41-PE antibodies. (**b**) Platelets were incubated with the same compounds, then stimulated with thrombin (50 mU/mL) for 2 min and analyzed by flow cytometry. Data are presented as means ± SD, non-parametric Mann–Whitney test, *n* = 6. *—*p* < 0.05 compared to control (C) sample. Control and thrombin (Ø) samples were treated with corresponding concentration of DMSO.

As mentioned above, only very limited information concerning the effects of bibenzyls on platelets is available. Therefore, we analyzed the effects of selected bibenzyls on platelet activation induced by thrombin. EN15 at a concentration of 60 µM strongly (up to 23 ± 12% compared to thrombin-induced activation taken as 100%) reduced integrin αIIbβ3 surface expression in platelets, (Figure 9a) which correlates with the inhibition of fibrinogen binding (Figure 9b). EN19, EN48, and erianin had no effect on thrombin-induced integrin αIIbβ3 surface expression and their activation (Figure 9a,b).

**Figure 10:**
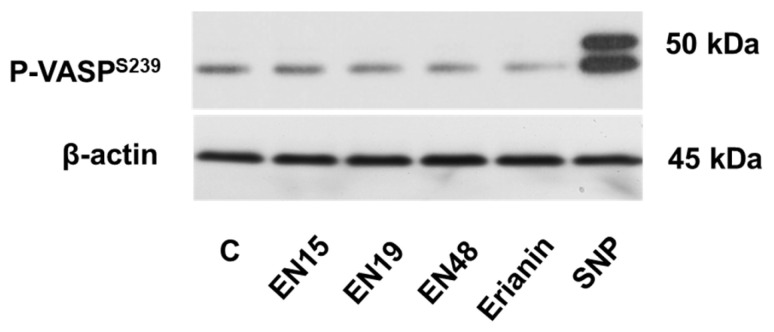
Bibenzyls do not induce VASP phosphorylation. Washed platelets were incubated with EN15/EN19/EN48 or erianin (all 60 µM) for 2 h at 37 °C. Sodium nitroprusside (SNP, 1 µM), which strongly increases VASP phosphorylation, was used as a positive control. Then, probes were processed for Western blot analysis with phospho-VASP (Ser239) antibodies. Actin served as a loading control. C—control treated with corresponding concentration of DMSO. A representative blot from three separate experiments is shown. Full blots are presented in the Supplementary Materials (Supplementary Figure S3).

Cyclic nucleotides (cAMP/cGMP) and corresponding protein kinases, protein kinase A (PKA) and protein kinase G (PKG), are the well-established mechanisms of platelet inhibition. Activation of these pathways can be monitored by the phosphorylation of their common substrate, vasodilator-stimulated phosphoprotein (VASP) [30]. None of the tested compounds induced VASP phosphorylation (Figure 10), indicating that these pathways are not involved in the inhibition of platelet activation.

**Figure 11:**
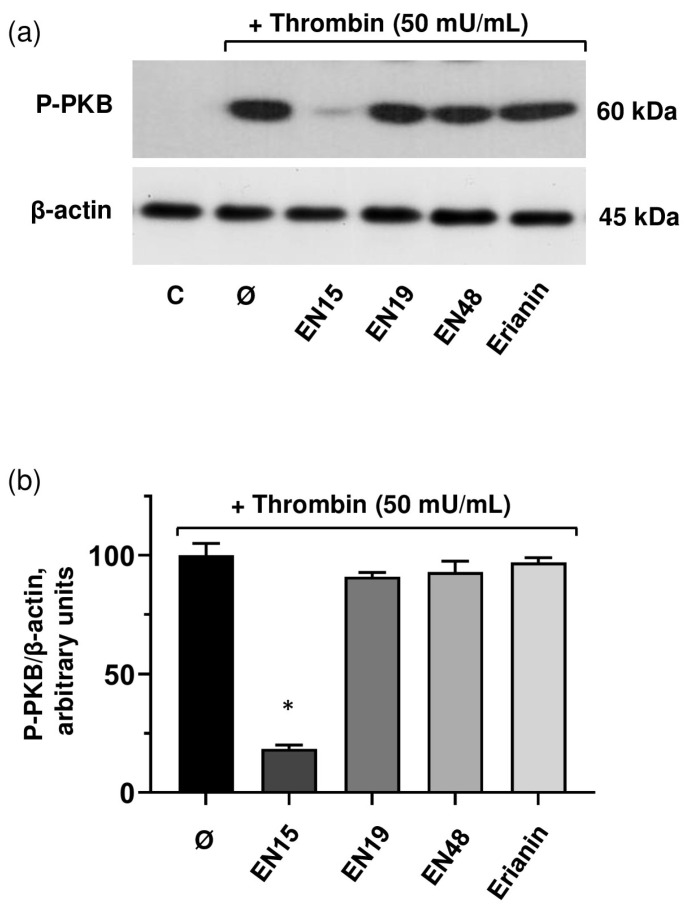
Among bibenzyls, only EN15 significantly decreases thrombin-induced PKB phosphorylation. (**a**) Platelets were incubated with EN15/EN19/EN48, or erianin (all 60 µM) for 2 h at 37 °C, then stimulated with thrombin (50 mU/mL) for 2 min and processed for Western blot analysis. Actin served as a loading control. (**b**) Blots were quantitated as described in the Figure 7. Data are presented as means ± SD of three separate experiments from three different donors. One-way ANOVA, Levene’s test *p* > 0.05 followed by Tukey’s HSD test. *—*p* < 0.05 compared to thrombin sample (Ø). Control and thrombin (Ø) samples were treated with corresponding concentration of DMSO. Representative blot from three separate experiments is shown. Full blots are presented in the Supplementary Materials (Supplementary Figure S4).

PI3K/PDK/PKB pathways play a significant role in platelet activation [31,32]. We tested whether the inhibitory effect of EN15 is connected with the inhibition of PKB (protein kinase B) activation. From the tested compounds, only EN15 strongly inhibited thrombin-induced PKB phosphorylation (Figure 11a,b), which corresponds to the inhibition of αIIbβ3 integrin surface expression and activation (Figure 9). These data indicate that the effects of EN15 in platelets are mediated by at least two independent pathways, namely by the activation of p38 MAP kinase that is involved in platelet viability and PS surface exposure, and by PKB pathways that are inhibited in thrombin-stimulated platelets. Other tested bibenzyls had no significant effects on platelet activation.

## 3. Discussion

Anticancer therapy is often accompanied by thrombocytopenia and/or thrombotic complications, and platelets play a key role in both these processes; therefore, it is important to include an analysis of platelets’ status in addition to the anticancer activity of new chemicals. Bibenzyls, natural compounds with a very simple skeleton, are now considered as molecules for use in cancer therapy. Therefore, in this study, we analyzed the effects of several bibenzyls on MOLT-3 leukemia cells and platelets. Among the studied bibenzyls, erianin is intensively characterized as an anticancer substance that inhibits cancer cell lines by activating diverse intracellular molecular mechanisms. Src kinase was found to be a target of erianin in the mechanisms of triple-negative breast cancer cell line inhibition by erianin [33]. In several gastric cancer cell lines and in gastric cancer models, erianin inhibits cancer growth and epithelial–mesenchymal transition by targeting the LKB1-SIK2/3-PARD3-signaling axis [34]. In human hepatoma HepG2 cells, erianin can selectively inhibit the activity of the pyruvate carboxylase-mediated Wnt/β-catenin pathway [35]. We did not find in the literature any information concerning the effects of erianin on MOLT-3 leukemia cells and platelets; therefore, one of the goals of our study was elucidation of erianin action on this cell line and platelets. Unexpectedly, we found that erianin, even at relatively high (30 µM) concentrations, only slightly induced PS surface exposure (Figure 2), reduced MOLT-3 cells viability (Figure 3a), and had no effect on platelets (Figure 4). Next, we compared four bibenzyls (three of them, including EN15, EN19, and EN48, isolated from *Empetrum nigrum*) and erianin, which are structurally similar and differ from erianin only by the absence of a methoxy group in position 4′ and the presence/absence of methoxy/hydroxy groups in positions 3′ 4, 5 and 3′. Interestingly, EN19 and EN48 had no significant effects on platelets and MOLT-3 cells, whereas EN15 induced strong annexin-V binding and reduced platelet viability. LDH release induced by the compound in the medium indicates a rupture of membrane integrity, which is observed in caspase 3-independent types of death. The inhibitors of apoptosis, pyroptosis, necroptosis, calpain-induced necrosis, and autophagy do not influence death induced by EN15 in MOLT-3 cells and platelets. EN15 also inhibited αIIbβ3 integrin surface expression and thrombin-induced platelet activation. EN15 also strongly activated the p38 MAP kinase pathway (Figure 7), and inhibition of this kinase significantly prevented the decrease in viability of both MOLT-3 cells (Figure 8a) and platelets (Figure 8b). This indicates that the effects of EN15 on these cells are, at least partly, mediated by the activation of p38 MAP kinase. These data demonstrate that p38 MAPK is associated with cellular responses in cancer [36]. For example, xanthohumol and celastrol induce paraptosis in many cancer cell lines, which is accompanied by p38 activation [37]. On the other hand, only EN15 strongly prevented thrombin-induced PKB phosphorylation, which correlates with the inhibition of platelet activation.

Considering that all tested compounds (EN15, EN19, EN48 and erianin) have similar structures but differ in bioactivity—ranging from comparatively inactive (EN19 and EN48) to showing significant activity (EN15 and erianin), it can be inferred that certain structural elements are necessary for the compound to show activity under the performed bioassays. The main structural features shared by both EN15 and erianin include a phenolic hydroxy group in position 3′ along with methoxy groups in positions 4 and 5. Substitution of the methoxy group in position 5 of EN15 for a hydroxy group (EN48) leads to loss of activity. The differences in both compounds’ activities can also be explained through their structures—erianin contains an additional methoxy group in position 4′ and a methoxy group instead of a phenolic hydroxy group in position 3. Since the structure of erianin differs from EN15 by two substituents, it is difficult to deduce which substituent exactly or if both of them together are responsible for the observed change in bioactivity. The synthesis/isolation and subsequent bioactivity tests of two additional compounds—3′-hydroxy-3′4,5-trimethoxybibenzyl (isoamoenylin) [38] and 3,3′-dihydroxy-4,5,4′-tetramethoxybibenzyl, which differ by only one functional group from both EN15 and erianin— are necessary to elucidate the exact effect of the hydroxyl/methoxy group in position 3 and the presence/absence of a methoxy group in position 4′ on the observed differences in these compounds’ bioactivities. Nonetheless, the observed structural differences make erianin less polar in comparison to EN15 and limit the ability of the molecule to form hydrogen bonds due to it containing one less polar phenolic hydroxy group, which along with other factors can significantly alter the compounds’ ability to interact with proteins. Most likely, these structural differences between erianin and EN15 are responsible for the greater biological activity of EN15 on the studied models in comparison to erianin.

## 4. Materials and Methods

The following chemicals were used in this study: Thrombin (Roche, Mannheim, Germany); sodium nitroprusside, SB202190, chloroquine, dimethylfumarate (Sigma-Aldrich, Munich, Germany); calpeptin (Abcam Inc., Waltham, MA, USA); erianin (Cayman Chemical, Ann Arbor, MI, USA); AC-YVAD-CHO (BIOZOL, Eching, Germany); Z-DEVD-FMF (Tocris Bioscience, Bristol, UK); phospho-PKB (# 4060), phospho-p38 (# 9216), and anti-actin (# 4970) antibodies (Cell Signaling, Frankfurt, Germany); phospho-vasodilator-stimulated phosphoprotein (VASP) S239 (Clone 16c2) antibodies (Nano Tools, Teningen, Germany); Fibrinogen-Alexa-Fluor 647, Calcein-AM (Molecular Probes, Gottingen, Germany); FITC-conjugated CD41P, Phycoerythrin-conjugated annexin-V (BD Bioscience, Heidelberg, Germany); horseradish peroxidase conjugated anti-rabbit or anti-mouse IgG (Amersham, Freiburg, Germany). 3,3′-dihydroxy-4,5-dimethoxybibenzyl designated as EN15, 3,5-dihydroxy-4-methoxybibenzyl (EN19) and 3,5,3′-trihydroxy-4-methoxybibenzyl (EN48) were isolated from *Empetrum nigrum* and characterized previously [21]. Structures of erianin and isolated compounds are presented in the Supplementary Figure S1.

### 4.1. Cell Culture

Human T-lymphoblastic cell line MOLT-3, RPMI-1640 medium, fetal calf serum and antibiotics streptomycin sulfate and penicillin G were from Biolot (Saint-Petersburg, Russia). MOLT-3 cells were cultured in RPMI 1640 medium supplemented with 10% heat-inactivated fetal calf serum, penicillin (25 IU/mL) and streptomycin (25 μg/mL) at 37 °C in 5% CO_2_. For MTT test, LDH assay and flow cytometry analysis, cells (8 × 10^4^) were seeded in 96-well plates.

### 4.2. Human Platelet Preparation

Healthy adult volunteers were used as blood donors. Our studies with human platelets were approved by the Independent Ethics Committee at Sechenov Institute RAS, Russia (protocol # 03–02 from 28 February 2024), in accordance with our institutional guidelines and the Declaration of Helsinki. Informed consent was obtained from all participants. Human platelets were prepared according to a previous description with small modifications [29]. Blood was collected into ACD solution (12 mM citric acid, 15 mM sodium citrate, 25 mM D-glucose, final concentrations) and EGTA (2 mM final concentration) and was centrifuged at 200× *g* for 10 min at room temperature (RT) to obtain platelet-rich plasma (PRP). PRP was centrifuged for 10 min at 430× *g* and the pelleted platelets were washed once in CGS buffer (120 mM sodium chloride, 12.9 mM trisodium citrate, 10 mM D-glucose, pH 6.5), and the platelet pellet was resuspended in HEPES buffer (150 mM sodium chloride, 5 mM potassium chloride, 1 mM magnesium chloride, 1 mM calcium chloride, 10 mM D-glucose, 10 mM HEPES, pH 7.4). Washed platelets were used for experiments after 15 min rest in a 37 °C water bath.

### 4.3. LDH Assay

LDH activity was measured spectrophotometrically in the culture media and lysates of the MOLT-3 cells and platelets at 340 nm by analyzing NADH reduction during the pyruvate–lactate transformation according to the manufacturer’s protocol of LDH-Vital kit (Vital Diagnostic SPB, Saint-Petersburg, Russia). LDH released from MOLT-3 cells and platelets treated with different compounds was calculated as a percentage of the total LDH concentration after lysis of cells by Triton-X 100 taken as 100%.

### 4.4. MTT Assay

The 3-(4,5-Dimethylthiazol-2-yl)-2,5-diphenyltetrazolium bromide (MTT) assay was performed to evaluate the cytotoxic effect of the studied bibenzyls on MOLT-3 cells. MTT was added to each well for 2 h then tested compounds were introduced for indicated concentrations/time. The purple formazan crystals were dissolved using a solubilization solution (20% SDS—50% dimethylformamide—HCl) overnight and absorbance at 570 nm was measured using a microplate reader (CLARIOstar Plus microplate reader, BMG Labtech, Ortenberg, Germany). The results are expressed as a percentage relative to control cells.

### 4.5. Flow Cytometry Analysis of Platelets

Flow cytometry analysis was performed using CytoFlex (Beckman Coulter, Brea, CA, USA). Data were analyzed by CytExpert Acquisition and Analysis Software Version 2.4 (Beckman Coulter), FACSDiva software v6.1.3 (BD Biosciences, San Jose, CA, USA) and FlowJo v10.0.7 (Becton Dickinson, Ashland, OR, USA).

### 4.6. Platelet αIIbβ3 Integrin Activation, Phosphatidylserine Surface Exposure, Cell Viability

For flow cytometry examination, platelets (0.5 × 10^8^ cells/mL) were incubated with CD41-PE antibody (1:30, 10 min) and were gated according to the size as CD41 positive. For the detection of surface phosphatidylserine (PS) or activated αIIbβ3 integrin, WP (30 µL) were labelled with annexin V-PE, fibrinogen-Alexa-647, respectively, for 15 min at RT before stimulation with studied compounds. The platelets were then diluted with annexin V-binding solution (140 mM NaCl, 10 mM HEPES, and 2.5 mM CaCl_2_) for annexin V or PBS for fibrinogen and immediately analyzed by flow cytometry. For determination of platelet viability, we used Calcein-AM but not MTT because the latter requires very long incubation time (2–3 h) and may be cytotoxic for platelets. Washed platelets were incubated with studied compounds for 2 h, then Calcein-AM (0.1 µM) was added for the last 15 min of incubation. After incubation, samples were diluted with PBS (1:10) and analyzed by flow cytometry.

### 4.7. MOLT-3 Cell Phosphatidylserine Surface Exposure

MOLT-3 cells (8 × 10^4^ cells/mL) were incubated with investigated compounds for 24 h at 37 °C. Then annexin-V-PE (diluted 1:100) was added for 10 min at 37 °C. The reaction was stopped by dilution with annexin-V binding solution (1:20) for 10 min for further flow cytometry analysis.

### 4.8. Western Blot Analysis

Western blots were performed as described previously [29]. Washed platelets (3 × 10^8^ platelets/mL) were treated with indicated compounds and after that they were lysed with Laemmli sample buffer. Proteins were separated by SDS-PAGE, transferred to nitrocellulose membranes. The membranes were incubated with appropriate primary antibodies overnight at 4 °C. For visualization of the proteins, goat anti-rabbit or anti-mouse IgG conjugated with horseradish peroxidase were used as secondary antibodies followed by ECL detection (GE Healthcare, Chicago, IL, USA). Blots were analyzed densitometrically using NIH Image J software v1.54g.

### 4.9. Data Analysis

Platelet experiments were performed on platelets from at least four different donors; data are presented as means ± SD using GraphPad Prism 8 (GraphPad Software, San Diego, CA, USA) for data analysis. The data were confirmed to follow a normal distribution by Shapiro–Wilk’s test (*p* > 0.05). Differences between groups were analyzed by one-way analysis of variance (ANOVA) with Tukey’s honestly significant difference (HSD) post hoc analysis, when the samples were homoscedastic (Levene’s test, *p* > 0.05). When equal variances were not assumed, Tamhane’s T2 post hoc analysis was used. For paired group analysis, Mann–Whitney U test was used. *p* < 0.05 was regarded statistically significant.

### 4.10. Bibenzyl Isolation and Structural Elucidation

Air-dried and powdered shoots of *E. nigrum* were repeatedly extracted through maceration with solvent (n-hexane, ethanol) until the fresh extracts’ coloration was diminished in comparison to the initially obtained extract. All of the obtained extracts were combined and concentrated under reduced pressure. The concentrated extracts were then dissolved in a minimal amount of solvent and fractionated using column chromatography on stationary phases with different selectivity (silica gel, Sephadex LH-20). The compounds EN15, EN19 and EN48 were isolated from the obtained fractions using preparative RP-HPLC. Structural elucidation was performed, based on the compounds NMR and HR-ESI-MS spectra as described in [21].

## 5. Conclusions

Bibenzyls with a very simple skeleton are now recognized as compounds for use in cancer therapy and many molecules from the bibenzyl group have shown promising anticancer activity. Therefore, the characterization of a new bibenzyl with a strong biological activity is important because it might be a candidate for developing new anticancer drugs. In this study, we presented the bibenzyl, 3,3′-dihydroxy-4,5-dimethoxybibenzyl (EN15), which possesses stronger biological activity than the well-studied erianin. We also showed that the p38 MAP kinase pathways are involved in the activity of EN15, and the prevention of thrombin-induced platelet activation is correlated with the inhibition of PKB.

## Data Availability

The data underlying this article will be shared at reasonable request to the corresponding author.

## Supplementary Materials

The following supporting information can be downloaded at: https://www.mdpi.com/article/10.3390/molecules30142965/s1, Supplementary Figure S1: Structure of the tested compounds; Supplementary Figure S2: Full blots of Figure 7a; Supplementary Figure S3: Full blots of Figure 10; Supplementary Figure S4: Full blots of Figure 11a.
