# Supplementary material for: Death of Leukemia Cells and Platelets Induced by 3,3′-Dihydroxy-4,5-Dimethoxybibenzyl Is Mediated by p38 Mitogen-Activated Protein Kinase Pathway
Source: Molecules. 2025 Jul 15;30(14):2965. doi: 10.3390/molecules30142965 (PMC12300253; doi:10.3390/molecules30142965)
Supplement: Supplementary file 1 [file molecules-30-02965-s001.zip › molecules-3703077-supplementary.pdf]

Supplementary Materials

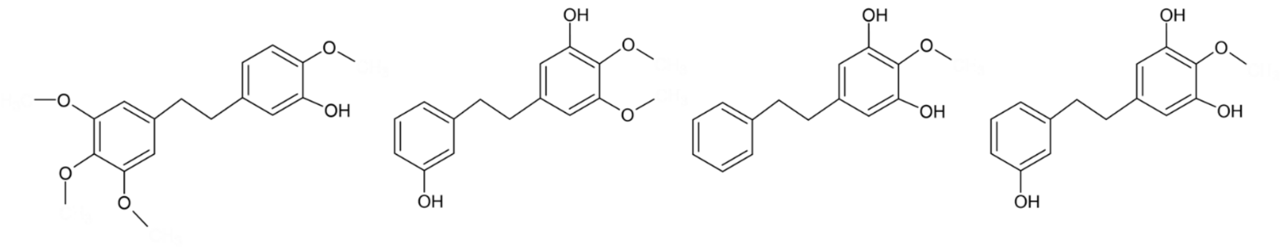

Erianin (3'-hydroxy-3,4,4',5-tetramethoxybibenzyl)

EN15 (3,3'-dihydroxy-4,5-dimethoxybibenzyl)

EN19 (3,5-dihydroxy-4-methoxybibenzyl)

EN48 (3,5 ,3'-trihydroxy-4 -methoxybibenzyl)

Supplementary Figure S1. Structure of the tested compounds

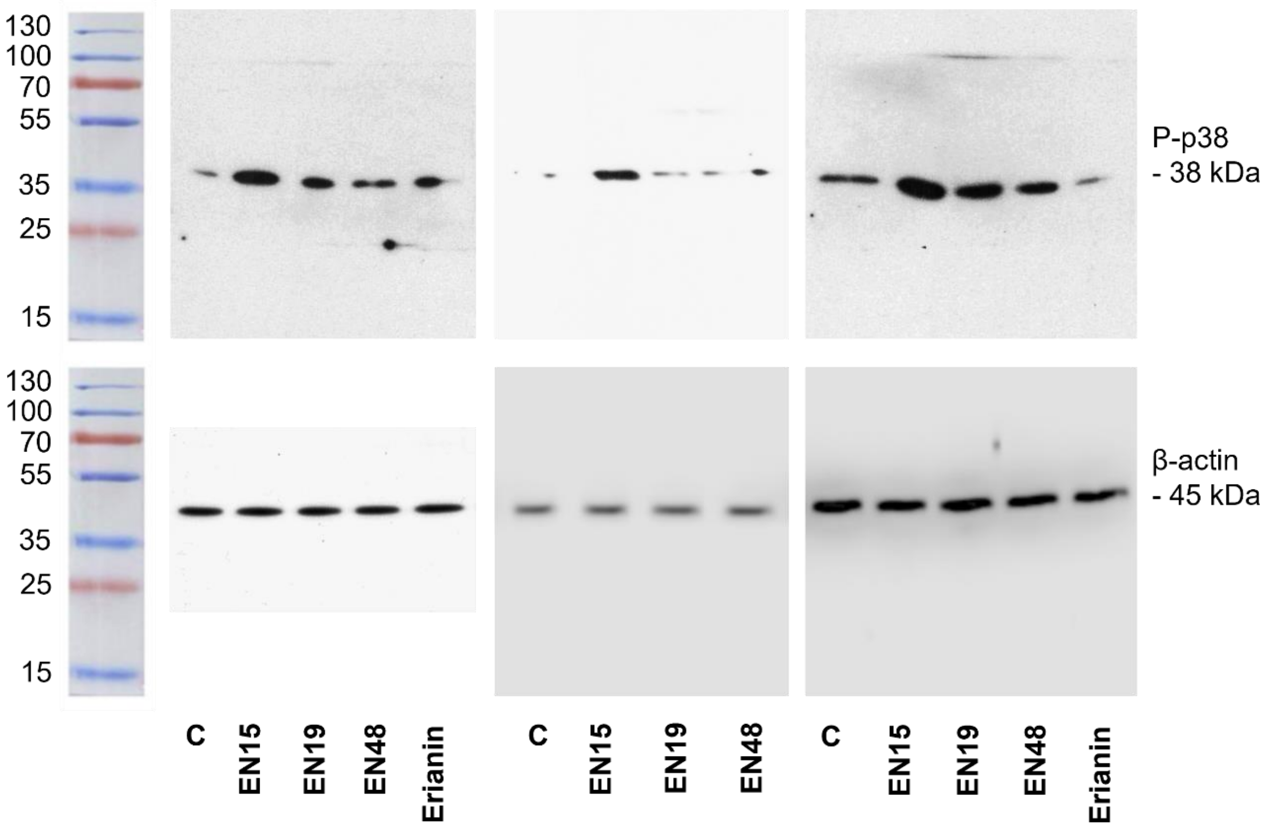

Supplementary Figure S2. Full blots of Figure 7a

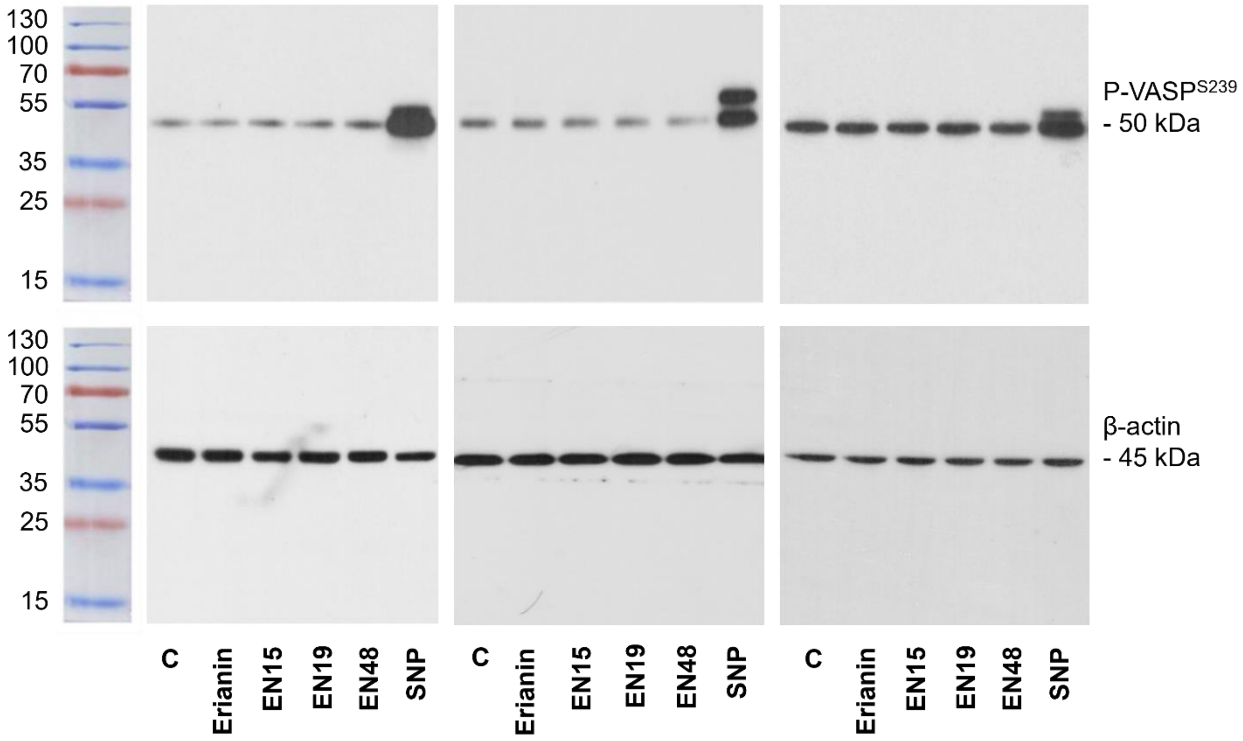

Supplementary Figure S3. Full blots of Figure 10

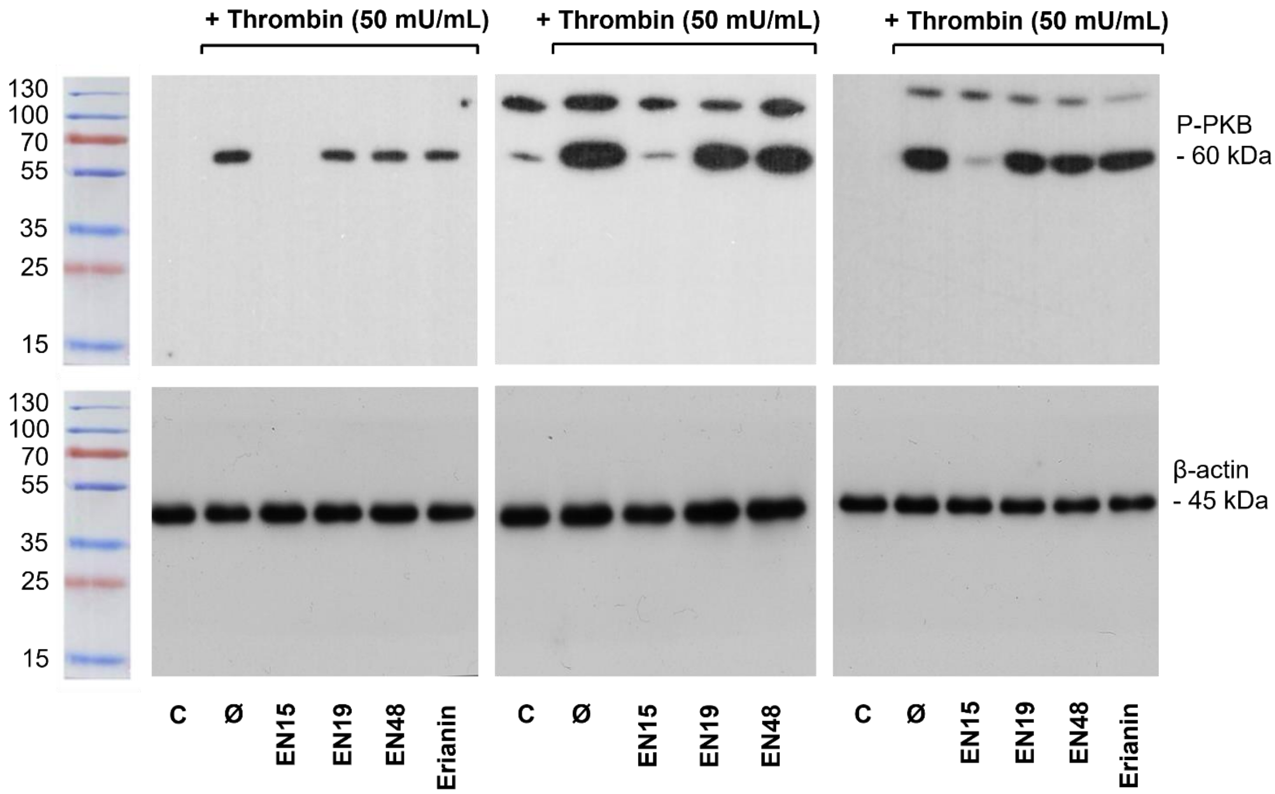

Supplementary Figure S4. Full blots of Figure 11a
